# Dark-field X-ray imaging for the assessment of osteoporosis in human lumbar spine specimens

**DOI:** 10.3389/fphys.2023.1217007

**Published:** 2023-07-17

**Authors:** Florian T. Gassert, Theresa Urban, Alexander Kufner, Manuela Frank, Georg C. Feuerriegel, Thomas Baum, Marcus R. Makowski, Christian Braun, Daniela Pfeiffer, Benedikt J. Schwaiger, Franz Pfeiffer, Alexandra S. Gersing

**Affiliations:** ^1^ Department of Diagnostic and Interventional Radiology, School of Medicine and Klinikum Rechts der Isar, Technical University of Munich, Munich, Germany; ^2^ Chair of Biomedical Physics, Department of Physics, School of Natural Sciences, Technical University of Munich, Garching, Germany; ^3^ Munich Institute of Biomedical Engineering, Technical University of Munich, Garching, Germany; ^4^ Department of Neuroradiology, Klinikum Rechts der Isar, School of Medicine, Technical University of Munich, Munich, Germany; ^5^ Institute of Forensic Medicine, University Hospital of Munich, LMU Munich, Munich, Germany; ^6^ Munich Institute for Advanced Study, Technical University of Munich, Garching, Germany; ^7^ Department of Neuroradiology, University Hospital of Munich, LMU Munich, Munich, Germany

**Keywords:** dark-field imaging, osteoporosis, spine, medical physics, bone

## Abstract

**Background:** Dark-field imaging is a novel imaging modality that allows for the assessment of material interfaces by exploiting the wave character of x-ray. While it has been extensively studied in chest imaging, only little is known about the modality for imaging other tissues. Therefore, the purpose of this study was to evaluate whether a clinical X-ray dark-field scanner prototype allows for the assessment of osteoporosis.

**Materials and methods:** In this prospective study we examined human cadaveric lumbar spine specimens (vertebral segments L2 to L4). We used a clinical prototype for dark-field radiography that yields both attenuation and dark-field images. All specimens were scanned in lateral orientation in vertical and horizontal position. All specimens were additionally imaged with CT as reference. Bone mineral density (BMD) values were derived from asynchronously calibrated quantitative CT measurements. Correlations between attenuation signal, dark-field signal and BMD were assessed using Spearman’s rank correlation coefficients. The capability of the dark-field signal for the detection of osteoporosis/osteopenia was evaluated with receiver operating characteristics (ROC) curve analysis.

**Results:** A total of 58 vertebrae from 20 human cadaveric spine specimens (mean age, 73 years ±13 [standard deviation]; 11 women) were studied. The dark-field signal was positively correlated with the BMD, both in vertical (r = 0.56, *p* < .001) and horizontal position (r = 0.43, *p* < .001). Also, the dark-field signal ratio was positively correlated with BMD (r = 0.30, *p* = .02). No correlation was found between the signal ratio of attenuation signal and BMD (r = 0.14, *p* = .29). For the differentiation between specimens with and without osteoporosis/osteopenia, the area under the ROC curve (AUC) was 0.80 for the dark-field signal in vertical position.

**Conclusion:** Dark-field imaging allows for the differentiation between spine specimens with and without osteoporosis/osteopenia and may therefore be a potential biomarker for bone stability.

## 1 Introduction

Osteoporosis is a metabolic bone disease leading to reduced bone strength and consequently to fragility fractures (Consensus Development Conference, 1993). Osteoporotic fractures, particularly in the spine or hip, are associated with a considerable reduction in quality of life, increased morbidity and mortality (Bliuc et al., 2009). Osteoporosis is also a socioeconomic challenge to our aging society and a major global health concern. The early diagnosis of osteoporosis remains challenging, therefore osteoporosis is often overlooked and existing effective prevention and medical treatment are not initiated in many patients (Khosla and Shane, 2016). Bone mineral density (BMD) is so far the single most important parameter for the assessment of bone strength (NIH Consensus Development Panel on Osteoporosis Prevention et al., 2001), and dual energy X-ray absorptiometry (DXA) is considered the standard of reference for clinical bone densitometry (Kanis, 2002). Yet, BMD measurements and the corresponding T-scores acquired using DXA have repeatedly shown to be insufficient (Schuit et al., 2004; Leonhardt et al., 2020). Quantitative CT (qCT) has been established as an equal alternative to DXA, showing a higher sensitivity for osteoporosis in previous studies (Li et al., 2013; Leonhardt et al., 2021). However, there are significant limitations to qCT, including lack of standardization, increased radiation exposure as well as increased costs (Choksi et al., 2018). Thus, other techniques have been introduced for the assessment of bone strength (Gluer et al., 2004; Leonhardt et al., 2021; Gassert et al., 2022a), such as grating-based X-ray dark-field vector radiography (Wen et al., 2009; Baum et al., 2015; Eggl et al., 2015).

Grating-based X-ray dark-field imaging (Pfeiffer et al., 2008) measures ultra-small-angle scattering that takes place at the material interfaces within the specimen under investigation (Willer et al., 2018), such as alveoli in the lung (Gassert et al., 2022b) or trabeculae in bones. With a clinical prototype for dark-field chest radiography, the techniques’ potential for the assessment of pulmonary diseases (Gassert et al., 2021; Willer et al., 2021; Gassert et al., 2022c; Gassert et al., 2022d; Gassert and Pfeiffer, 2022; Urban et al., 2022) has recently been demonstrated. While these previous studies evaluated the dark-field signal of the alveolar structure in chest radiography, this study aims to evaluate the dark-field signal of the trabecular bone structure. Due to the low dark-field signal of osseous structures in dark-field chest radiographs (Gassert et al., 2021), a higher setup sensitivity is necessary. This can be achieved by adjusting the sample position in the clinical dark-field prototype. A big advantage of grating-based X-ray dark-field imaging is that both a dark-field image and an attenuation-based image are acquired simultaneously in one single acquisition. The acquired attenuation-based image is similar to a conventional radiograph acquired with commercially available X-ray machines (Kattau et al., 2023). DXA also uses attenuation information, however, in a dual-energy mode.

Based on findings in previous studies with smaller specimens (Baum et al., 2015; Eggl et al., 2015), we hypothesize that a reduced number of trabeculae in lumbar vertebrae of specimens with osteoporosis or osteopenia results in a lower dark-field signal intensity. Also, as the dark-field prototype scanner is sensitive only to material interfaces in horizontal orientation we evaluate dark-field signal behavior in both horizontal and vertical orientation.

Therefore, the purpose of this study was to evaluate whether a clinical X-ray dark-field prototype enables the assessment of osteoporosis in lumbar spine specimens.

## 2 Methods

### 2.1 Specimen

Institutional review board approval was obtained prior to this study (Ethics Commission of the Medical Faculty, Technical University of Munich, Germany, Reference Number 70/17S). This study was conducted in accordance with the Declaration of Helsinki. Lumbar spine specimens were harvested (vertebral level L2 to L4) from human cadavers within 24 h after death. Inclusion criteria was a clinically indicated post-mortem examination. Exclusion criteria were previous spine surgery and known osseous metastatic disease. For one specimen, only L2 and L3 were harvested, resulting in a total of 59 vertebrae from 20 human cadaveric spine specimens.

### 2.2 CT imaging and BMD measurements

CT was performed on one dual-layer dual-energy CT (IQon Spectral CT, Philips Healthcare) with the following parameters: Collimation, 0.6 mm; pixel spacing, 0.3 mm; spiral pitch factor, 0.39; tube voltage (peak), 120 kV; tube current, 347 mA. BMD values were derived from asynchronously calibrated quantitative CT examinations (Roski et al., 2019; Roski et al., 2021). Hounsfield unit (HU) values were measured for all lumbar vertebrae in representative median slices using the IDS7 PACS (Sectra AB, Linkoeping, Sweden). Regions of interest (ROIs) were manually segmented by a radiologist (FTG, 4 years of experience in musculoskeletal imaging) in the sagittal plane in the anterior part of the vertebra and BMD values were then calculated from the average HU values, as described previously (Loffler et al., 2019). BMD values <120 mg/cm ^3^ were considered as osteoporotic/osteopenic, BMD values ≥120 mg/cm^3^ were considered normal (American College of Radiology, 2013).

### 2.3 X-ray dark-field imaging

**FIGURE 1:**
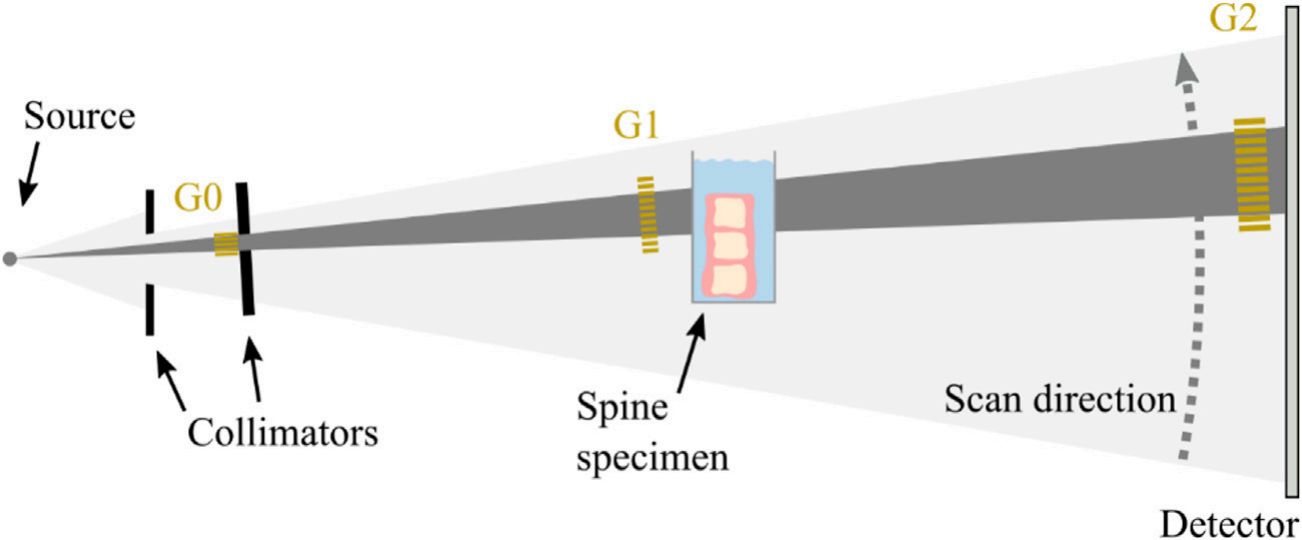
Schematic of the clinical prototype for dark-field radiography. The spine specimen is imaged in lateral orientation in a water bath close to the grating G1 in vertical position (as shown) as well as in horizontal position (not shown).

We used the prototype for clinical dark-field chest radiography described in (23). It consists of conventional medical X-ray devices (tube, MRC 200 0508 ROT-GS 1003, Philips Medical Systems, Hamburg, Germany, detector, PIXIUM 4343 F^4^, Trixell, Moirans, France) in combination with a Talbot-Lau interferometer with three gratings. From each acquisition, both attenuation and dark-field image can be reconstructed. To increase the setup’s sensitivity for assessment of osseous structure, we chose a larger distance between sample and analyzer grating than for patients in previous studies (Figure 1). Specimen were imaged in lateral orientation in a water bath to reduce potential effects of air around the sample. To reduce the influence of Compton scatter and beam hardening, the water container without the specimen was used in the reference scans, and a beam hardening correction was applied using aluminum as equivalent absorber material (Donath et al., 2009). All reported quantitative values for attenuation and dark-field signal are relative to water. Due to the one-dimensional, horizontal structure of the gratings, the setup is only sensitive to structural elements parallel to the grating lamella, i.e., in horizontal direction. As bones contain trabecular structures in both craniocaudal and lateral directions, the dark-field signal generated by the specimen depends on specimen orientation (Baum et al., 2015; Eggl et al., 2015). Specimens were therefore scanned in both, a vertical standing position (sensitive to trabecular structures in lateral orientation) as well as in a horizontal lying position (sensitive to trabecular structures in craniocaudal orientation).

### 2.4 Quantitative image evaluation

**FIGURE 2:**
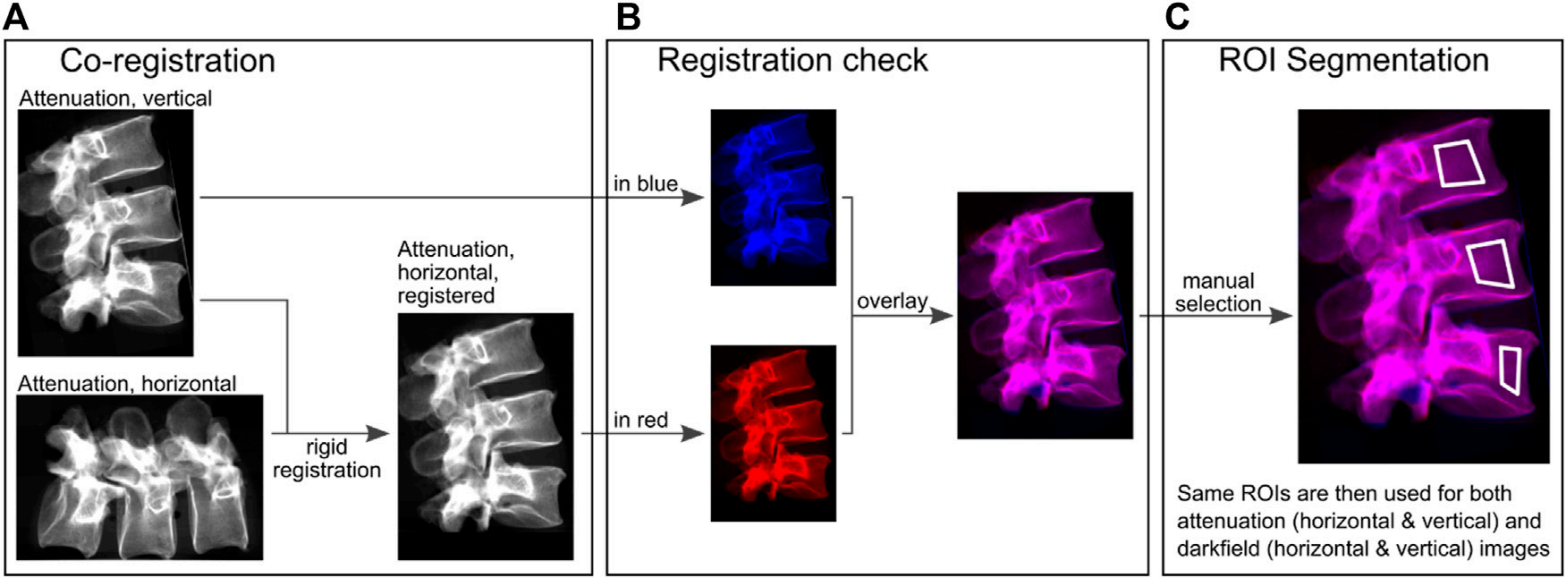
Quantitative image evaluation. **(A)**, attenuation images were used for rigid co-registration of vertical and horizontal images. **(B)**, these registrations were checked for consistency with color-coded overlays. Red (acquisition in horizontal orientation) and blue (acquisition in vertical orientation) correspond to the individual images and magenta corresponds to the same values after the images were superimposed on each other in order to check the registration. **(C)**, ROIs were manually selected in these overlays, excluding superimposition caused by transverse processes or sclerosis.

Attenuation images in horizontal and vertical position were co-registered with a rigid registration using Python and SimpleElastix (Figure 2A). ROIs were segmented manually in the anterior part of the vertebra using overlay images of horizontal and co-registered vertical attenuation images (Figure 2C). Areas with sclerosis and superimposition were not included in the segmentations. The same ROIs were applied to all attenuation and dark-field images of one spine specimen. Quantitative values were calculated from the mean signal within each ROI.

### 2.5 Statistical analysis

Statistical analysis was performed using Python, specifically NumPy (Harris et al., 2020) and SciPy (Virtanen et al., 2020). For detection of positioning inaccuracies, we tested the ratio of attenuation signal in horizontal and vertical position for outliers with Grubbs outlier test, leading to the exclusion of one vertebra. Accordingly, one vertebra was excluded from quantitative analysis. We tested for correlations between attenuation and dark-field signals in horizontal and vertical position and BMD using Spearman correlation statistics. Additionally, we also tested the ratio of signal in vertical and horizontal position for correlation with BMD. Differences in attenuation and dark-field signal in horizontal and vertical orientation were tested using the paired Wilcoxon test. Differences in attenuation and dark-field signal of vertebrae with and without osteoporosis/osteopenia (BMD <120 mg/cm^3^) were tested using Wilcoxon Mann-Whitney *U* test. The utility of the dark-field signal for prediction of osteoporosis/osteopenia was assessed using receiver-operating-characteristic (ROC) curves and the respective area under the curve (AUC). A significance level of .05 was used for all tests.

## 3 Results

### 3.1 Specimens

We studied 58 vertebrae from spine specimens from 20 donors (9 men, 11 women). The average age of the donors was 73 years ±13 [standard deviation], the average weight was 87 kg ± 29, and the average height was 165 cm ± 8 (Table 1).

**TABLE 1:** Specimen donor demographics.

Parameter	All	Healthy	Osteoporotic/Osteopenic		*p*-Value
Number of specimens	20	11	9		
Men/Women	9/11	6/5	3/6	.34	
Age (years)	73 ± 13	69 ± 14	76 ± 12	.15	
Weight (kg)	87 ± 29	97 ± 30	74 ± 22	.07	
Height (cm)	165 ± 8	166 ± 7	163 ± 10	.48	
BMI (kg/m^2^)	32 ± 10	35 ± 12	27 ± 6	.06	

Values are given as mean ± standard deviation. *p*-values for the significance of differences between the group with normal BMD, values and the osteoporotic/osteopenic group are listed in the very right column. Of the 9 specimens in the osteoporotic/osteopenic group, two consisted of two osteoporotic/osteopenic vertebrae and one healthy vertebra each.

### 3.2 Quantitative analysis

**FIGURE 3:**
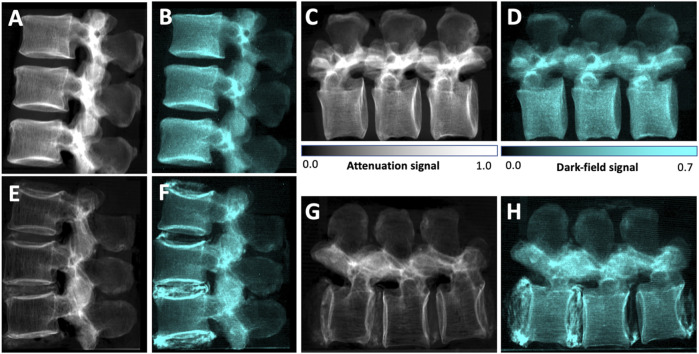
Lateral conventional **(A, C, E, G)** and co-registered dark-field **(B, D, F, H)** radiographs in vertical **(A, B, E, F)** and horizontal **(C, D, G, H)** position of the spine specimen of a 61-year-old man with normal BMD values (BMD = 224 mg/cm^3^) **(A–D)** and of a 58-year-old woman with osteoporotic BMD values (BMD = 63 mg/cm^3^) **(E–H)**. Compared to the specimen without osteoporosis/osteopenia, the attenuation signal appears reduced in the osteoporotic specimen. Also, the trabecular texture can be seen in both attenuation images, while it seems rarefied in the osteoporotic specimen. In the dark-field images, the signal of the trabecular bone is also lower in the osteoporotic specimen, however the signal difference is less distinct. The trabecular structure that can be seen in the attenuation images cannot be seen in the dark-field images, and if it is detected in the dark-field images, it appears to by very indistinct. In the osteoporotic specimen, calcifications within the intervertebral discs result in both an attenuation signal as well as a strong dark-field signal.

Figure 3 shows example attenuation radiographs and dark-field radiographs. All values are given as mean ± standard deviation. The average BMD across all vertebrae was 141 ± 59 mg/cm^3^. It was 75 ± 19 mg/cm^3^ in the osteoporotic/osteopenic subgroup (*n* = 23) and 184 ± 28 mg/cm^3^ in the group without osteoporosis/osteopenia (*n* = 35). For 11 specimens all vertebrae were osteoporotic/osteopenic (33 vertebrae) and for 7 specimens all vertebrae were non-osteoporotic/osteopenic (19 vertebrae, one was excluded, one was not harvested, see above). In two specimens two vertebrae were osteoporotic/osteopenic and one was non-osteoporotic/osteopenic each. The average mean dark-field signal across all vertebrae increased from 0.27 ± 0.06 in vertical position to 0.32 ± 0.06 in horizontal position (*p* < .001), the average mean attenuation signal was 0.27 ± 0.09 in both vertical position and horizontal position (*p* = .98).

**FIGURE 4:**
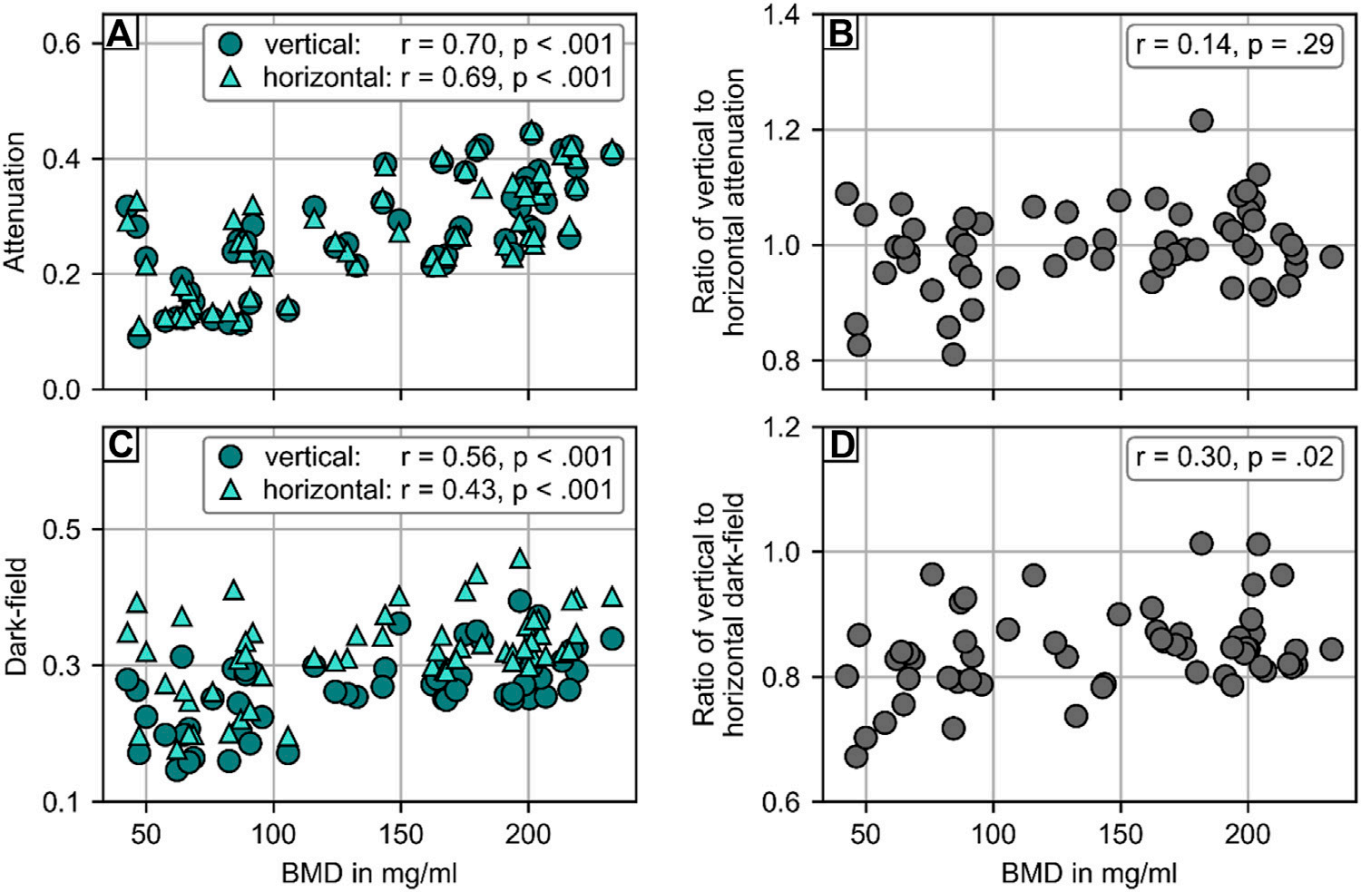
Statistical analysis of the attenuation and dark-field signal of 58 vertebrae from 20 spine specimen. **(A)** Comparison of attenuation signal with opportunistic BMD from CT. There was strong correlation between BMD and attenuation signal in both vertical (r = .74, *p* < .001) and horizontal position (r = .75, *p* < .001). **(B)** No correlation (r = .07, *p* = .62) was found for the ratio of attenuation signal in vertical and horizontal position with BMD. **(C)** Comparison of dark-field signal with BMD from CT. There was moderate correlation between BMD and attenuation signal in both vertical (r = .57, *p* < .001) and horizontal position (r = .51, *p* < .001). **(D)** Comparison of the ratio of dark-field signal in vertical and horizontal position and BMD. There was a weak correlation (r = .37, *p* = .005) between the ratio of dark-field signal in vertical and horizontal position and BMD.

The attenuation signal was positively correlated with the BMD, both in vertical (r = 0.70, *p* < .001) and horizontal position (r = 0.69, *p* < .001; Figure 4). The same applied for the dark-field signal (vertical: r = 0.56, *p* < .001; horizontal: r = 0.43, *p* < .001). When calculating signal ratios between vertical and horizontal position, the average ratio was 1.00 ± 0.07 for the attenuation signal and 0.84 ± 0.07 for the dark-field signal. No correlation was found between the signal ratio of attenuation signal and BMD (r = 0.14, *p* = .29). The dark-field signal ratio, however, was positively correlated with BMD (r = 0.30, *p* = .02).

**FIGURE 5:**
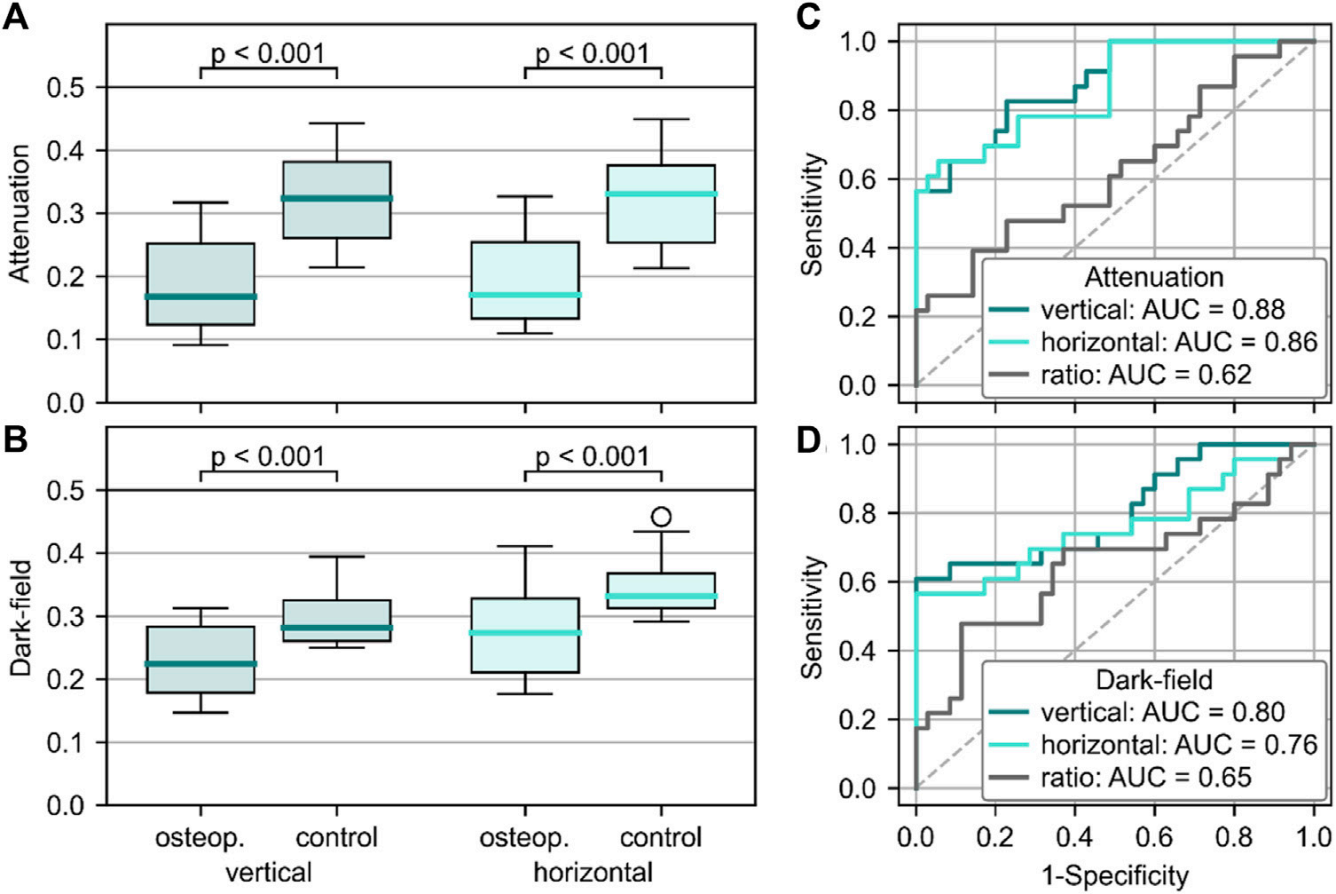
Quantitative analysis of the ability to differentiate between osteoporotic/osteopenic vertebrae and those with normal BMD. Signal intensities were higher in osteoporotic/osteopenic vertebrae when compared to healthy controls for both attenuation **(A)** and dark-field signal **(B)**, regardless of the positioning (*p* < .001 for all). Receiver operating characteristic curve showed the capability of attenuation **(C)** and dark-field **(D)** signal to differentiate between osteoporotic/osteopenic spine specimen and such with normal BMD with the highest AUC for signal in vertical positioning (attenuation: 0.88, dark-field: 0.80).

Figure 5 shows the quantitative evaluation for the differentiation between vertebrae with and without osteoporosis/osteopenia. The mean attenuation signal in both vertical (0.19 ± 0.07) and horizontal (0.20 ± 0.07) orientation of vertebrae with osteoporosis/osteopenia was significantly lower than that of controls (vertical: 0.32 ± 0.07, *p* < .001; horizontal: 0.32 ± 0.07, *p* < .001). The average dark-field signal was also significantly lower in vertebrae with osteoporosis/osteopenia for both vertical (0.23 ± 0.05 vs. 0.29 ± 0.04, *p* < .001) and horizontal orientation (0.28 ± 0.7 vs. 0.34 ± 0.04, *p* < .001). For the differentiation between vertebrae with and without osteoporosis/osteopenia, the area under the receiver operating characteristic curve (AUC) was 0.88 for attenuation signal in vertical position, 0.86 for attenuation signal in horizontal position, 0.80 for dark-field signal in vertical position, 0.76 for dark-field signal in horizontal position, and 0.65 for ratio of dark-field signal in vertical to horizontal position. The optimum cutoff value in dark-field signal in vertical scanning position was .24, the respective Youden index was J = 1.61. Applying this cutoff value for the differentiation between specimens with and without osteoporosis/osteopenia, sensitivity, specificity, and accuracy were 0.61, 1.00, and 0.83, respectively.

## 4 Discussion

In this prospective study, we evaluated whether a clinical prototype for dark-field radiography enables the assessment of osteoporosis in human cadaveric spine specimens. Both the attenuation signal and the dark-field signal were positively correlated with the BMD, both in vertical and in horizontal position. The ratio of dark-field signal in vertical and horizontal scanning position was also positively correlated with BMD (r = 0.30, *p* = 0.02), while the ratio for attenuation signal showed no correlation with BMD (r = 0.14, *p* = 0.29). For the differentiation between specimens with and without osteoporosis/osteopenia, the AUC was 0.80 for dark-field signal in vertical position with an optimum cutoff value of .24, yielding a sensitivity, specificity, and accuracy of 0.61, 1.00, and .84, respectively.

X-ray dark-field imaging is an imaging technique that has recently reached the human scale. It takes advantage of the wave properties of X-rays, as material interfaces generate small-angle scattering resulting in dark-field signal (Pfeiffer et al., 2008). Previous studies (Gassert et al., 2021; Willer et al., 2021; Frank et al., 2022; Urban et al., 2022) have focused on the investigation of dark-field imaging in the lungs as the numerous alveoli with their countless tissue-air-interfaces cause a high signal intensity. In these studies, the settings were chosen in a way that bone generates hardly any signal in order to reduce overlay artifacts. However, dark-field signal intensity can be increased significantly when positioning the object under examination closer to the phase grating of the Talbot-Lau-interferometer (Donath et al., 2009). In this study, this effect was utilized by placing the specimen much closer to the phase grating G1. In this setting, dark-field signal is assumed to be generated by the material interfaces of the trabecular structure in the spongy bone. The lower dark-field signal in patients with lower BMD most likely results from a lower number of material interfaces due to a lower trabecular number, which is well in line with the fact that trabecular number is reduced in patients with osteoporosis/osteopenia (Amling et al., 1996; Leonhardt et al., 2021).

Another important finding results from the fact that our prototype scanner is sensitive to material interfaces only in horizontal position, that is in the orientation of the gratings of the Talbot-Lau-Interferometer. Thus, in order to evaluate whether dark-field signal indeed is generated by interfaces between trabecula and bone marrow, we scanned the specimens both in vertical and horizontal position. The results demonstrated that the dark-field signal was higher in the horizontal position compared to the vertical position, suggesting a higher number of trabeculae in vertical orientation compared to horizontal orientation, which is in line with previous findings (Amling et al., 1996; Homminga et al., 2004; Matsuura et al., 2008). As expected, the respective attenuation signal did not change with different positioning, yet strongly correlating with the BMD in both positions. When calculating the ratio between dark-field signal in vertical and horizontal position, we found a positive correlation with BMD, meaning that the ratio is lower in osteoporotic/osteopenic patients, suggesting a lower trabecular number in these patients. This effect is stronger for trabeculae in horizontal orientation, which is in line with previous studies showing that mainly the horizontally aligned trabeculae show a reduction in thickness and number within osteoporotic bone (Amling et al., 1996; Homminga et al., 2004; Matsuura et al., 2008; Eggl et al., 2015). The remaining vertically aligned trabeculae tend to maintain their thickness and number (Thomsen et al., 2002; Chen et al., 2008; Chen et al., 2013). This underlines, that dark-field signal may indeed serve as a biomarker for material interfaces such as the trabecular structure of the spongy bone.

When calculating the AUC for the differentiation between specimens with and without osteoporosis/osteopenia, the AUC values for attenuation signal were higher compared to dark-field signal intensities. This was expected, since the attenuation signal is directly proportional to the specimen density, which can also be quantified with the BMD measurements in osteoporotic and non-osteoporotic vertebrae, which served as standard of reference in this study. Yet, in an *in-vivo* patient setting, however, the attenuation of the vertebra would be superimposed with the attenuation of surrounding tissue, which is the reason that conventional attenuation cannot be used for BMD determination. Currently in clinical routine DXA, a dual-energy technique, is used as reference standard for the BMD assessment. DXA measurements are mainly acquired in an anterior-posterior projection due to the lower effective dose compared to a lateral acquisition. Nevertheless, previous studies have shown DXA measurements to be insufficient for BMD determination due to, e.g., superimposition of degenerated posterior elements of the vertebrae (Li et al., 2013). Since dark-field imaging is formed through the mechanism of small-angle scattering, dark-field imaging may obtain complementary structural information about the trabecular bone microstructure at the micrometer length scale without requiring high-resolution detectors, which are not applicable in the medical field due to the dose requirements for patients. Dark-field also may suffer less from superimposition caused by surrounding tissues, due to a specific sensitivity to material interfaces in horizontal orientation (trabecula orientation effected most due to osteoporosis) and since soft tissue does not generate any dark-field signal. Moreover, obtaining very detailed information of the trabecular bone by using dark-field x-ray imaging in addition to DXA-based BMD measurements may increase the sensitivity and specificity for osteoporosis compared to DXA alone while exposing the patient to lower effective doses compared to qCT-based BMD measurements. Therefore, this approach may be imaginable in the future, yet, *in vivo* studies are needed for further investigation.

This is the first study to evaluate dark-field imaging for the assessment of osteoporosis in a human sized scanner prototype. Our results are consistent with Eggl et al., who previously found a higher anisotropy in osteoporotic vertebrae, apposite to a changing trabecular structure in a much smaller number of osteoporotic specimens (Eggl et al., 2015). Moreover, this previous study was performed using an experimental dark-field set up.

This study has limitations. First, we did not correct for different sample thicknesses influencing the measured signals. Second, we used qCT measurements for the quantification of BMD and not DXA measurements, since robust DXA measurements were insufficient in the spine specimens. This was due to the *ex-vivo* experimental setting lacking surrounding tissue. Also, BMD measurement from CT have previously shown to be a more reliable method for the assessment of bone stability (Link, 2012; Li et al., 2013; Loffler et al., 2019). Third, while this study was conducted *ex-vivo* with only little surrounding tissue, the results need to be further investigated in vivo studies in the future.

In conclusion, a prototype dark-field x-ray imaging system indeed yields a measurable signal from trabeculae within human lumbar specimens, and allows the differentiation between vertebrae with and without osteoporosis/osteopenia. This suggests that the information on trabecular bone assessed using dark field x-ray imaging may complement the information obtained on bone mineral density and therefore may increase the diagnostic confidence in osteoporotic patients.

## Data Availability

The raw data supporting the conclusion of this article will be made available by the authors, without undue reservation.
